# Gliptins: A New Class of Oral Antidiabetic Agents

**DOI:** 10.4103/0250-474X.59541

**Published:** 2009

**Authors:** K. G. Seshadri, M. H. B. Kirubha

**Affiliations:** Department of Endocrinology, Diabetes and Metabolism, Sri Ramachandra University, Porur, Chennai-600 116, India

**Keywords:** Dipeptidyl peptidase-4 (DPP-4) inhibitors, glucagon like peptide-1 (GLP-1), sitagliptin, Type 2 Diabetes Mellitus

## Abstract

India has the largest population of patients with type 2 diabetes mellitus. The conventional agents used to treat type 2 diabetes frequently exhibit reduced efficacy over time leading to inadequate glycaemic control and are also associated with adverse effects. Hence, there is a need for alternative therapies that can overcome the limitations associated with conventional antidiabetic agents. This review focuses on Gliptins, which have become a research area of intense focus and present an alternative therapeutic strategy for patients with type 2 diabetes. Gliptins show significant improvements in glycaemic control and are well tolerated, particularly with regard to weight change and hypoglycemia. Hence, gliptins are considered as useful agents for the treatment of type 2 diabetes mellitus.

The prevalence of Type 2 Diabetes Mellitus (DM) is increasing all over the world, especially in South Asia. India has largest population of diabetic patients. The International Diabetes Federation (IDF) estimates the number of people with diabetes in India will reach 80 million by the year 2025.

There are at least seven different classes of agents used as monotherapy, or in combinations for the treatment of diabetes mellitus. These include metformin, sulphonylureas, meglinitides, alpha-glucosidase inhibitors, thiazolidinediones (TZD), glucagon like peptide-1 (GLP-1) agonists and insulin[1,2]. Many conventional agents frequently exhibit reduced efficacy over time, leading to inadequate glycaemic control. Several of these agents are also associated with adverse effects that include weight gain, hypoglycemia and gastrointestinal distress[2]. There is a need therefore, for alternative therapies that can overcome the limitations associated with conventional anti-hyperglycemic medications.

## Gut peptides and diabetes:

In 1902, Bayliss and Starling first hypothesized that the gut might directly signal the pancreas. In 1930, the term incretin was first used to describe the enhanced glucose lowering effect that was seen when a gut extract was fed to dogs. In the 1960s, Perley and Kipnis demonstrated that almost twice as much as insulin was released when glucose was infused directly into the gut rather than into the blood as an IV solution. This discovery renewed interest in a search for compounds produced by the gut that could lower blood glucose levels[3].

## The incretins, GLP-1 and GIP:

The two major incretin hormones are glucose-dependent insulinotropic polypeptide (GIP) and glucagon like peptide (GLP-1). Both GIP and GLP-1 promotes pancreatic β cell growth and survival. GIP is a 42 amino acid peptide secreted from the K cells of the duodenum. As GIP contains an alanine at position 2, it is an excellent substrate for dipeptidyl peptidase-4 (DPP-4), an essential enzyme regulating the degradation of GIP. Full length GIP (1-42) is rapidly converted to bioinactive GIP (3-42) within minutes of secretion from the gut K cell. Hence, circulating immunoreactive GIP represents a mixture of active GIP (1-42) and inactive GIP (3-42).

GLP-1 is a product of glucagon gene. Proglucagon is processed to glicentin, oxyntomodulin, GLP-1 and GLP-2 in gut L cells of the ileum and colon via processing that requires prohormone convertase-1. Bioactive GLP-1 is generated from GLP-1 (1-37) and exists as two equipotent circulating molecular forms GLP-1 (7-37) and GLP-1 (7-36) amide. Release occurs rapidly after eating, with carbohydrate, fat and protein each acting as stimulants to secretion[4]. The major (≈80%) circulating form of GLP-1 is GLP-1(7-36) amide. Compared with GLP-1 (7-37), GLP-1(1-37) has a 100 fold lower affinity for the GLP-1 receptor[5].

## Dipeptidyl peptidase-4 (DPP-4):

DPP-4, also known as CD26, is a cell surface glycoprotein with peptidase activity that is present in the blood and most tissues. DPP-4 was first reported in 1966 and is essential for the control of GLP-1 bioactivity and glucose homeostasis. DPP-4 is a complex molecule that exists in a membrane-bound form on the endothelial surfaces and as a soluble form in the circulation; both forms have proteolytic activity. DPP-4 readily inactivates the insulinotropic hormone GLP-1[1,5–8]. Native GLP-1 is rapidly degraded with a very short plasma half life of 6.1±0.8 min for GLP-1 (7-37) and 5.3±0.4 min for GLP-1 (7-36) amide in healthy subjects[5]. Both GLP-1 (7-37) and GLP-1 (7-36) amide contain an alanine at position 2 and are rapidly degraded by DPP-4 to GLP-1 (9-36) amine or GLP-1 (9-37) within about 5-6 min following release from the gut L cells[9].

## Therapeutic potential of GLP-1 in diabetes mellitus:

In individuals with normal glucose metabolism, postprandial glucose levels stimulate the release of GLP-1 and GIP from the gut. These peptides regulate the release of insulin and glucagon from the pancreas and this process is termed the incretin effect[10]. In patients with type 2 DM, postprandial GLP-1 secretion is modestly impaired but GLP-1 actions are preserved. While, GIP secretion is normal in type 2 diabetics, these individuals are relatively resistant to the acute insulinotropic effect of exogenous GIP administration. The effect of GLP-1 on insulin secretion is dependent on plasma glucose concentration, with a greater insulin secretory effect at higher glucose levels and a minimal effect at euglycemic levels. Consequently, therapies that potentiate endogenous incretin action appear to have a low risk of hypoglycemia[1,2,4,5,7–9,11]. Two major approaches used to achieve adequate active levels of GLP-1 have been incretinmimetics (GLP-1 agonists) and DPP-4 inhibitors.

## Physiological effects of GLP-1:

**Fig. 1:**
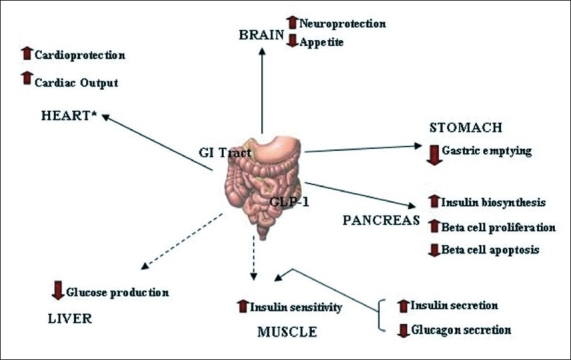
Actions of GLP-1 *Postulated. GLP-1 has been reported to exert direct actions on the pancreas, heart, stomach and brain, and indirect actions on liver and muscles (adopted from[9]). (→)Indirect action, (→) Direct action.

GLP-1 has direct effects on the endocrine pancreas, heart, stomach and brain and indirect effects on the liver and muscle (fig. 1)[9]. GLP-1 stimulates insulin secretion and decreases glucagon secretion. It also improves insulin sensitivity and enhances glucose disposal. GLP-1 also delays gastric emptying, induce satiety and in the pharmacologic doses decreases body weight. In animal and *in vitro* studies it has been shown to increase beta cell mass. It has also been hypothesized to have beneficial cardiovascular and CNS effects[5].

## GLP-1 and its agonists:

Natural GLP-1 has little benefit because it is broken down by DPP-4. This has led to the development of new drug that is not broken down as quickly. Exenatide is a synthetic exendin-4 and is the first GLP-1 based therapy approved for human use in April 2005 in the United States. Exenatide was approved in July 2007 in India[12]. Exendin-4 is a naturally occurring component of the saliva of the Gila monster (*Heloderma suspectum*), a large lizard native to the south western US and shares 53% sequence identity with GLP-1; it is resistant to DPP-4 degradation[3,13].

Exenatide is similar in its structure to GLP-1 but resists breakdown in the body and thus lasts much longer. Both an increase in beta cell mass in rats and improvement of first phase insulin release toward normal have been seen with this drug. This action is hypothesized to delay progression of type 2 DM. Exenatide is indicated for therapy of patients with type 2 DM inadequately controlled on metformin, sulfonylurea, thiazolidinedione or combination of the two. Studies of combination treatment with metformin and exenatide have shown little risk of hypoglycemia. People on exenatide eat about 20% less and often lose weight. With prolonged use, weight loss has been associated with improvements in blood pressure and lipids[3,4,8,12].

The recommended dose of exenatide is 5-10 μg twice daily. Exenatide therapy reduces HbA1_c_ levels by approximately 1% and body weight by 2-3 kg. The most common adverse effect is nausea[1,4,5,7,8,13,14]. At the time of writing, the US FDA has received 36 reports of pancreatitis in patients taking exenatide, two of whom died because of hemorrhagic or necrotizing pancreatitis[15]. Other new GLP-1 agonists currently in clinical trials are summarized in Table 1[4,6,14]. GLP-1 may prove to have a therapeutic role in heart disease, with small-scale human trials showing that it improves left ventricular function, both after primary angioplasty for acute myocardial infarction and in patients with New York Heart Association Class III/IV heart failure[4].

**TABLE 1:** GLP-1 AGONISTS AVAILABLE AND IN DEVELOPMENT

Drug	Highest Development Phase	Company
Exenatide	Launched	Eli Lilly
Exenatide-LAR	Phase 3	Amylin, Alkermes, Eli Lilly
Liraglutide	Pre-registration	Novo Nordisk
CJC 1134	Phase 2	Conju Chem. Biotechnologies
Albiglutide	Phase 2	Human Genome sciences
Taspoglutide	Phase 2	Roche, Ipsen
AVE 0010	Phase 3	Zealand Pharma
R-51077	Phase 2	Ipsen, Roche and Teijin

## DPP-4 inhibitors:

Inhibition of DPP-4 by DPP-4 inhibitors enhances the hormone activity of GLP-1 and other bioactive peptides (GIP, gastrin releasing peptide), thereby stimulating the release of insulin and reducing the secretion of glucagons. This effect contributes to the regulation of elevated blood glucose levels in type 2 DM patients[5,7]. Proof-of-concept for the efficacy of DPP-4 inhibitors as antidiabetic agents in humans was reported using NVP DPP728, a first generation small molecule DPP-4 inhibitor, which further encouraged the discovery and development of such agents[1].

DPP-4 inhibitors have become a research area of intense focus with a number of pharmaceutical companies involved in the development of a new diabetes therapy. As of this writing, there are more than 20 different DPP-4 inhibitors being developed for various therapeutic interests-mainly type 2 DM. Although, a number of DPP-4 inhibitors have been described, all have limitations relating to potency, stability or toxicity. Sitagliptin and vildagliptin are two DPP-4 inhibitors that have been approved in India for human use in October 2007 and January 2008, respectively[12]. Other new DPP-4 inhibitors currently in clinical trials are summarized in Table 2[6]. The DPP-4 inhibitors improve metabolic control without causing severe hypoglycemia. DPP-4 inhibitors tend to be weight neutral[5].

**TABLE 2:** DPP-4 INHIBITORS AVAILABLE AND IN DEVELOPMENT

Drug	Highest Development Phase	Company
Sitagliptin	Launched	Merck & Co
Sitagliptin+Metformin	Launched	Merck & Co
Vildagliptin	Launched	Novartis
Vildagliptin+Metformin	Launched	Novartis
Saxagliptin	Launched	Bristol Myers Squibb
Saxagliptin+Metformin	Phase 3	Bristol Myers Squibb
Alogliptin	Pre-registration	Takeda
BI 1356 BS	Phase 2	Boehringer Ingelheim
Melogliptin	Phase 2	Glenmark Pharmaceuticals Ltd.
AMG 222	Phase 2	Amgen
MP 513	Phase 2	Mitsubishi Tanabe Pharma Corporation
PHX 1149	Phase 2	Phenomix Corporation
PSN 9301	Phase 2	Prosidion
R 1579	Phase 2	Roche
SYR 472	Phase 2	Takeda San Diego
TA 6666	Phase 2	Mitsubishi Tanabe Pharma Corporation
Denagliptin	Phase 3	Glaxo Smithkline

## Sitagliptin:

Sitagliptin produces sustained inhibition of DPP-4 and is indicated for the treatment of type 2 DM to improve glycaemic control in combination with metformin or a TZD, when diet and exercise, plus metformin or a TZD do not provide adequate glycaemic control. Sitagliptin can be used either as monotherapy or in combination with metformin. It should not be used in patients with type 1 diabetes or to treat diabetic ketoacidosis[1,11,16–18]. Sitagliptin 50 mg and metformin 500/1000 mg as combination therapy received approval in India in April 2008[12].

The recommended dose of sitagliptin is 100 mg once daily, with or without food[16]. Sitagliptin reduces HbA_1c_ level approximately by 0.7% and is similarly effective when combined with metformin or pioglitazone. Fasting plasma glucose was reduced with DPP-4 inhibitors compared with placebo, with sitagliptin appearing to be more effective than vildagliptin[17]. The most common side effects that may occur with sitagliptin are upper respiratory tract infection, nasopharyngitis and headache. Occasionally, it may cause stomach discomfort and diarrhea. The main restriction involves reducing the dose in patients with significant kidney insufficiency[16,18].

## Dosage adjustment of sitagliptin in patients with renal insufficiency:

For patients with mild renal insufficiency in which the creatinine clearance (CrCl) is ≥ 50 ml/min, approximately corresponding to serum creatinine levels of ≤1.7 mg/dl in men and ≤1.5 mg/dl in women, no dosage adjustment for sitagliptin is required. For patients with moderate renal insufficiency (CrCl 30 to < 50 ml/min, approximately corresponding to serum creatinine levels of >1.7 to ≤3.0 mg/dl in men and >1.5 to ≤2.5 mg/dl in women), the dose of sitagliptin is 50 mg once daily. For patients with severe renal insufficiency (CrCl < 30 ml/min, approximately corresponding to serum creatinine levels of >3.0 mg/dl in men and >2.5 mg/dl in women) or with end-stage renal disease (ESRD) requiring hemodialysis or peritoneal dialysis, the dose of sitagliptin is 25 mg once daily. Sitagliptin may be administered without regard to the timing of hemodialysis. Because there is a need for dosage adjustment based upon renal function, assessment of renal function is recommended prior to initiation of sitagliptin and periodically thereafter. Creatinine clearance can be estimated from serum creatinine using the Cockcroft-Gault formula[16].

## Clinical studies with sitagliptin:

Clinical studies have demonstrated that sitagliptin is safe and efficacious for the management of hyperglycaemia in type 2 DM. The first monotherapy study (n=741, duration-24 w) demonstrated that sitagliptin administrated in 100 mg and 200 mg daily doses reduced HbA_1c_ levels by 0.79 and 0.94% respectively, at 24 w. These differences were statistically significant when compared to placebo (p<0.001). The proportion of patients achieving a mean HbA_1c_ <7% was 41, 45 and 17% for the sitagliptin 100 mg, 200 mg and the placebo groups, respectively (P<0.001 for sitagliptin vs. placebo)[19].

The second monotherapy study (n=521, duration-18 w) demonstrated that sitagliptin administrated in 100 mg and 200 mg daily doses reduced HbA_1c_ levels by 0.60 and 0.48%, respectively. The proportion of patients achieving a mean HbA_1c_ <7% was 35.8, 28.6 and 15.5% for the sitagliptin 100 mg, 200 mg and placebo groups, respectively[20]. In another report, daily doses of 100 mg sitagliptin were associated with statistically significant (p<0.001) 0.65% reductions in HbA_1c_ in patients with an initial mean HbA_1c_ of 8%[21].

Seven randomized, double-blind clinical trials evaluated the efficacy and safety of sitagliptin in combination with other antidiabetic agents for patients with type 2 DM who had inadequate glycaemic control, HbA_1c_ ≥6.5% and <11% despite treatment. The main efficacy measure in all studies was the change in HbA_1c_ from baseline. Secondary endpoints included; proportion of patients achieving HbA_1c_ <7 %[17].

Goldstein *et al.* (n=1,091; duration-24 w) compared initial treatment with sitagliptin 100 mg/d combined with metformin (1 g or 2 g/d) with sitagliptin alone, metformin alone or placebo. All active treatments produced significant reductions in HbA_1c_ from baseline compared to placebo (0.83% for sitagliptin alone, 0.99 and 1.3% for metformin 1 g and 2 g, and 1.57 and 2.07% for sitagliptin plus 1 g and 2 g metformin, respectively, p<0.001 for combination vs. respective monotherapy). Significantly more patients treated with combination therapy achieved HbA_1c_ values <7% compared with patients treated with sitagliptin monotherapy or metformin monotherapy (p<0.01)[22].

Rosenstock *et al.* (n=353; duration-24 w) compared the efficacy of sitagliptin with placebo added to ongoing pioglitazone therapy (30 or 45 mg/d). A significantly greater proportion of patients achieved an HbA_1c_ <7% with sitagliptin plus pioglitazone group than with placebo plus piglitazone group (45.4% Vs 23%, p<0.001). Significantly fewer patients receiving sitagliptin required rescue therapy (6.9% Vs 14.0%, p<0.05)[23]. A 52 w non-inferiority trial (n=1,172) compared the addition of sitagliptin or glipizide up to 10 mg bid to metformin (>1500 mg/d) therapy. Sitagliptin plus metformin was shown to be non-inferior to glipizide plus metformin in reducing HbA_1c_ (-0.67% for both groups). The proportion of patients achieving an HbA_1c_ <7% was similar between the two groups (63% Vs 59%). Sitagliptin was associated with a small weight loss (-1.5 kg) compared to a small weight gain (+1.1 kg) in those receiving glipizide in this study (p<0.001). However, more patients in the sitagliptin group withdrew from the study (mainly due to lack of efficacy: 15% Vs 10%) compared with the glipizide group[24].

Three randomized controlled trials (n=1,164; duration 18 to 30 w) compared sitagliptin with placebo in patients receiving ongoing metformin (≥1500 mg/d) therapy. The addition of sitagliptin reduced HbA_1c_ levels to a greater extent than the addition of placebo (between treatment difference of -51 to -1.0%, p<0.001). More patients in the sitagliptin plus metformin group achieved an HbA_1c_ <7% than in the placebo plus metformin groups (22 to 55% vs. 3 to 38%, p< 0.05)[25–27]. A 24-w, double-blind randomized controlled trial (n=441) compared sitagliptin with placebo in patients receiving ongoing glimepiride (≥4 mg/d) therapy with or without metformin (≥1500 mg/d). The addition of sitagliptin reduced HbA_1c_ levels to a greater extent than the addition of placebo (between treatment difference of -0.74%, p<0.001). More patients treated with sitagliptin achieved an HbA_1c_ <7% than patients treated with placebo (17% vs. 5%, p<0.001)[28].

## Safety considerations:

DPP-4 has effects beyond its proteolytic action, including T-cell activation and proliferation. Dipeptidyl peptidase encompasses a large family of enzymes. Full-scale inhibition of an enzyme system such as this could cause myriad deleterious effects, and, because of this, selectivity is also an important issue with DPP-4 inhibitors. DPP-4 is structurally and functionally related to other enzymes including DPP-8 and DPP-9. Inhibition of closely related DPP-8 and DPP-9 enzymes has been associated with severe toxicity in animal studies. Hence, there is some concern that prolonged inhibition of DPP-4 activity or off-target actions with non-selective inhibitors could lead to adverse side effects. Sitagliptin exhibits a >2,600-fold higher affinity for DPP-4 than for DPP-8 and DPP-9 enzymes[2,5].

Sitagliptin is a pregnancy risk category B agent and should be used during pregnancy if deemed necessary. Caution is also advised in women who are nursing. It is currently unknown whether sitagliptin is secreted in human breast milk, and the effects on nursing babies are also unknown. Safety and efficacy in patients <18 y of age have not been studied[7].

## Vildagliptin:

Vildagliptin is the second DPP-4 inhibitor approved for human use and is indicated for the control of hyperglycemia in patients with type 2 DM. Vildagliptin produces sustained inhibition of DPP-4 when administered and produces moderate increases in GLP-1 and GIP. Vildagliptin is available as 50 mg and 100 mg tablets with a recommended dose of 50 mg once daily if used in combination with metformin or a TZD and 50 mg once daily if used in combination with a sulfonylurea[18]. Vildagliptin as a fixed dose combination product containing vildagliptin 50 mg with metformin hydrochloride 500/850/1000 mg received approval in India in July 2008[12]. The most common adverse events with vildagliptin are mild and include nasopharyngitis, headache and dizziness[8].

Oral vildagliptin in combination with metformin, a sulfonylurea or a TZD improved glycaemic control in adults with type 2 DM. In trials in patients with diabetes inadequately controlled with metformin, vildagliptin provided an additional reduction in HbA_1_ c levels of 1.1% and was shown to be as effective as pioglitazone as add-on therapy in a non-inferiority trial[29]. Vildagliptin was found to be weight neutral and generally well tolerated with low risk of hypoglycemia. Further investigation is required to accurately position vildagliptin relative to other, well established anti-diabetic agents.

## CONCLUSIONS

Incretin based therapies present an alternative therapeutic strategy for patients with type 2 diabetes and, in general, show significant improvements in glycemic control and are well tolerated, particularly with regard to weight change and hypoglycemia. In addition, this class may preserve or even reverse the decline in β cell function that is observed in patients with diabetes. These characteristics suggest that gliptins should be considered useful agents in monotherapy and combination therapy for the treatment of type 2 diabetes.
